# The inhibition of mammalian target of rapamycin (mTOR) in improving inflammatory response after traumatic brain injury

**DOI:** 10.1111/jcmm.16702

**Published:** 2021-07-10

**Authors:** Michela Campolo, Giovanna Casili, Marika Lanza, Alessia Filippone, Marika Cordaro, Alessio Ardizzone, Sarah Adriana Scuderi, Salvatore Cuzzocrea, Emanuela Esposito, Irene Paterniti

**Affiliations:** ^1^ Department of Chemical Biological, Pharmacological and Environmental Sciences University of Messina Messina Italy; ^2^ Department of Pharmacological and Physiological Science Saint Louis University School of Medicine St Louis MO USA

**Keywords:** apoptosis, astrogliosis, behavioural performance, KU0063794, mTOR, mTORC1, mTORC2, neuroinflammation, neuronal death, rapamycin, traumatic brain injury

## Abstract

Traumatic brain injury (TBI) provokes primary and secondary damage on endothelium and brain parenchyma, leading neurons die rapidly by necrosis. The mammalian target of rapamycin signalling pathway (mTOR) manages numerous aspects of cellular growth, and it is up‐regulated after moderate to severe traumatic brain injury (TBI). Currently, the significance of this increased signalling event for the recovery of brain function is unclear; therefore, we used two different selective inhibitors of mTOR activity to discover the functional role of mTOR inhibition in a mouse model of TBI performed by a controlled cortical impact injury (CCI). Treatment with KU0063794, a dual mTORC1 and mTORC2 inhibitor, and with rapamycin as well‐known inhibitor of mTOR, was performed 1 and 4 hours subsequent to TBI. Results proved that mTOR inhibitors, especially KU0063794, significantly improved cognitive and motor recovery after TBI, reducing lesion volumes. Also, treatment with mTOR inhibitors ameliorated the neuroinflammation associated with TBI, showing a diminished neuronal death and astrogliosis after trauma. Our findings propose that the involvement of selective mTORC1/2 inhibitor may represent a therapeutic strategy to improve recovery after brain trauma.

## 1 INTRODUCTION

Traumatic brain injury (TBI) is a severe health problem affecting many people, contributing to a substantial amount of deaths and cases of disability. The view that neurological, behavioural and cognitive deficits are well‐known sequelae of TBI, leading to functional impairment and a worsening in quality of life, is widespread.^1^

Brain damage occurs from a direct neuronal tissue damage to the inflammatory response and excitotoxic damage that initiates secondary injury, characterized by microglial and astrocytes activation, resulting in the release of reactive oxygen species and pro‐inflammatory mediators.^2^, ^3^, ^4^ These responses may regulate the microglial cell activity leading to the clearance of tissue debris and to the subsequent resolution of the inflammatory response. Furthermore, recent study recognized the autophagy mechanism, mediated by phosphatidylinositol/protein kinase B Akt/mammalian target of rapamycin (PI3K/Akt/mTOR) pathway, a central role in brain responses associated with head trauma.^5^, ^6^ Autophagy is a major actor in the control of cell size, dendrite and axon outgrowth during brain development, and repair.^7^, ^8^ Numerous molecular components contribute to autophagy induction and progression, like the mammalian target of rapamycin (mTOR) as well as Beclin 1 and LC3. The two mTOR complexes, mTOR complex‐1 (mTORC1) and mTOR complex‐2 (mTORC2), play a crucial role in the control of cell proliferation.^9^, ^10^ Both have distinctive downstream targets: different biological functions and, importantly, different sensitivity to the drug rapamycin. Growth factors and hormones typically stimulate this pathway through the activation of receptor tyrosine kinases, leading to the PI3K activation and Akt downstream, which in turn regulates the activity of several signalling molecules, including mTOR.

Previously, we showed that mTOR inhibition produced a neuroprotective effect in an in vivo murine model of spinal cord injury (SCI), where the inflammatory response is the key contributor of expansion of the lesion and further loss of neurologic function.^11^ Moreover, it is known that mTORC1 inhibition, mediated by treatment with rapamycin, has protective effects in animal models of TBI in terms of behavioural performance measured by extent of tissue damage, motor function and neurological score.^12^, ^13^

Although different in vivo studies demonstrated the positive effects of rapamycin on behavioural performance, limitations for its use in the human patient population exist, as rapamycin produces considerable side effects, especially when treatment is prolonged.^14^ Based on these results, in this study we investigated a new second generation of mTOR inhibitor, such as KU0063794, in an animal model of TBI. KU0063794 confers specific inhibition on both mTORC1 and mTORC2, suggesting that this double inhibition may be a valid approach to improve functional recovery after TBI.

## 2 MATERIALS AND METHODS

### 2.1 Animals

Male CD1 mice (20‐25 g, Envigo), aged between 8 and 10 weeks, were used for all studies. Mice were located in a controlled location with standard rodent chow and water. Animals were kept at 22 ± 1℃ with a 12‐hour light, 12‐hour dark cycle. This study was approved by the University of Messina Review Board for the care of animals, in compliance with Italian regulations on protection of animals (n° 617/2017‐PR released on 02/08/2017). Animal care was in accordance with Italian regulations on the use of animals for the experiment (DM116192) as well as with EEC regulations (OJ of EC L 358/1 12/18/1986).

### 2.2 Controlled cortical impact (CCI) experimental TBI

The animals were anaesthetized, and TBI was induced by a controlled cortical impact (CCI) as previously described.^15^

### 2.3 Experimental design

Mice were arbitrarily allocated into the following groups:

Sham + vehicle: mice were subjected to the surgical procedures, without the application of impact tip, and vehicle dimethyl sulfoxide (DMSO) was administered at 1 and 4 hours after craniotomy (n = 20);

TBI + vehicle: mice were subjected to CCI, and vehicle (DMSO) was administered at 1 and 4 hours after craniotomy (n = 20);

TBI + rapamycin (1 mg/kg): mice were subjected to CCI, and rapamycin (1 mg/kg in 10% DMSO) was administered orally 1 and 4 hours after craniotomy (n = 20);

TBI + KU0063794 (1 mg/kg): mice were subjected to CCI, and KU0063794 (1 mg/kg in 10% DMSO) was administered intraperitoneally i.p. 1 and 4 hours after craniotomy (n = 20).

Sham + rapamycin (1 mg/kg): mice were subjected to the surgical procedures without the application of impact tip, and rapamycin (1 mg/kg) was administered orally at 1 and 4 hours after craniotomy(n = 20).

Sham + KU0063794 (1 mg/kg): mice were subjected to the surgical procedures without the application of impact tip, and KU0063794 (1 mg/kg) was administered i.p. at 1 and 4 hours after craniotomy (n = 20).

The choice of using two different treatment modalities, intraperitoneal administration for KU0063794 and oral administration for rapamycin, is based on previous studies in our laboratory^11^ and in the bibliography.^16^, ^17^

As describe below, mice (n = 20 from each group and 10 for each technique) were killed at 24 h after TBI to evaluate the various parameters.

In order to reach the minimum number of mice required for every technique, an ANOVA (fixed effects, omnibus, one‐way) was defined ‘a priori’ with the G‐power software. This statistical test supplies a professional method to analyse the sample size required to make the experiments.

### 2.4 Behavioural testing

In another experimental set, all animals were subjected to behavioural tests, performed during the light cycle phase and in enclosed behaviour rooms (50‐55 dB ambient noise) within the housing room. Three different reliable expert observers, blinded to the damage status, led the experiment. The following tests were performed:

#### 2.4.1 Rotarod test

The rotarod treadmill (Accuscan, Inc.) provided a motor balance and coordination assessment. This test was executed as previously described.^18^

#### 2.4.2 Elevated biased swing test

The EBST provided a motor asymmetry parameter and involved handling the animal by its tail and recording the direction of the biased body swings. The EBST was performed as previous described.^18^

#### 2.4.3 Tail suspension test

The tail suspension test (TST) was conducted as previously described.^19^ The method is based on the observation that a mouse suspended by the tail shows alternate periods of agitation and immobility. Mice were securely fastened by the distal end of the tail to a flat stick surface and suspended in a visually isolated. The presence or absence of immobility, defined as the absence of limb movement, was sampled over a 6‐minute test session.

### 2.5 Tissue processing and histological analysis

Coronal sections of 7 μm thickness from the perilesional brain area of each animal were evaluated. Damaged neurons were counted, and the histopathologic changes of the grey matter were scored on a six‐point scale.^18^ The scores from all the sections of each brain were averaged to give a final score for individual mice. All the histological studies were performed in a blinded fashion.

### 2.6 Quantification of lesion volume

After kill, brains were cut into 5 coronal slices of 2 mm thickness by using a McIlwain tissue chopper (Campden Instruments Ltd.) and incubated in 2% solution of 2,3,5‐triphenyltetrazolium chloride (TTC; Sigma‐Aldrich). Infracted area and volume were calculated from digital images (Canon 4×, Canon Inc.) and ImageJ software 36. The measure of lesion volume and area was performed on coronal brain slices for a total of three slices per animal.

### 2.7 Immunohistochemistry

Brain tissue containing the lesion (1 cm on each side of the lesion) was fixed in 10% (w/v) buffered formaldehyde 24 hours after TBI and sliced in 7‐μm sections for paraffin‐embedding previously described.^15^ Afterwards, the sections were incubated overnight with one of the following primary antibodies diluted in PBS: anti‐GFAP (1:500, sc‐33673), anti‐IBA1 (1:500, sc:32725), anti‐TNF‐α (1:500, sc‐52746), anti‐IL1β (1:500, sc:32294), anti‐Bax (1:500, sc:526) and anti‐Bcl2 (1:500, sc:492). The immunohistochemical images were collected by Zeiss microscope using AxioVision software. For graphic representation of densitometric analyses, we measured the intensity of positive staining (brown staining) by computer‐assisted colour image analysis (Leica QWin V3). The percentage area of immunoreactivity (determined by the number of positive pixels) was expressed as per cent of total tissue area (red staining) as seen previously.^20^

### 2.8 Western blot analyses for IκB‐α, NF‐κBp65, COX‐2, iNOS, Bax, Bcl‐2 and p‐mTOR

Western blot was performed on samples harvested 24 hours post‐TBI. Cytosolic and nuclear extracts were prepared as described previously.^21^ The membranes were probed with specific Abs: anti‐NF‐κBp65 (1:500; sc:8008), anti‐iNOS (1:500; sc:8310), anti‐IκB‐α (1:500; sc:1643), anti‐COX2 (1:500; sc‐1746), anti‐IκB‐α (1:500; sc:1643), anti‐phospho‐mTOR (1:1000; #2971S), anti‐Bax (1:500 sc:7480) and anti‐Bcl‐2 (1:500 sc:7382), in 1 × PBS, 5% w/v non‐fat dried milk, 0.1% Tween‐20 at 4℃, overnight. β‐actin protein (cytosolic fraction 1:500; sc:8432) or lamin A/C (nuclear fraction 1:500 Sigma‐Aldrich Corp.) was used to samples normalization. Signals were detected with enhanced chemiluminescence (ECL) detection system reagent according to the manufacturer's instructions (Thermo). The relative expression of the protein bands was quantified using a standardization to β‐actin and lamin A/C levels. Images of blot signals (8 bit/600 dpi resolution) were imported to analysis software (Image Quant TL, v2003).

### 2.9 Mouse Romo1 (Reactive oxygen species modulator 1) ELISA Kit

ELISA kit assay for Romo1 (Reactive oxygen species modulator 1) was performed on brain samples. In details, brain tissue homogenates were weighted and homogenized in PBS enriched with protease inhibitors. The homogenates were then centrifuged for 5 minutes at 5000× *g* to get the supernatant. The total protein concentration was determined, and the samples were mixed with the kit reagents for 30 minutes at 37℃. The ROMO1 expression was evaluated reading the OD absorbance at 450 nm in microplate reader. The following kit, for mouse protein identifications, was used: Mouse Romo1 (Reactive oxygen species modulator 1, Catalogue No.: EM0699, Wuhan Fine Biotech).

### 2.10 TUNEL staining

TUNEL was performed using a ‘Fluorescein In Situ Cell Death Detection Kit’ (Roche Diagnostics GmbH), according to the manufacturer's instructions, as previously described,^22^ to define the level of intracerebral neuronal cell death.

### 2.11 Materials

Rapamycin and KU0063794 were purchased by Tocris Bioscience (98%). All stock solutions were made in non‐pyrogenic saline (0.9% NaCl, Baxter) or 10% dimethyl sulfoxide (DMSO). Except otherwise stated, all compounds were obtained from Sigma‐Aldrich Company Ltd.

### 2.12 Statistical evaluation

All values in the figures and text are expressed as mean ± *SE* of the mean (SEM) of N number of animals. In histology or immunohistochemistry experiments, the images exhibited are representative of at least three experiments performed on different days. Results were analysed by one‐way ANOVA followed by a Bonferroni post hoc test for multiple comparisons. A *P*‐value <.05 was considered significant. For one‐way ANOVA statistic test, a single ‘*F*’ value indicated as variation between sample means/variation within the samples was shown.

## 3 RESULTS

### 3.1 KU0063794 treatment reduced the degree of brain trauma and the infarct outcome, modulating neurological and motor deficit

**FIGURE 1:**
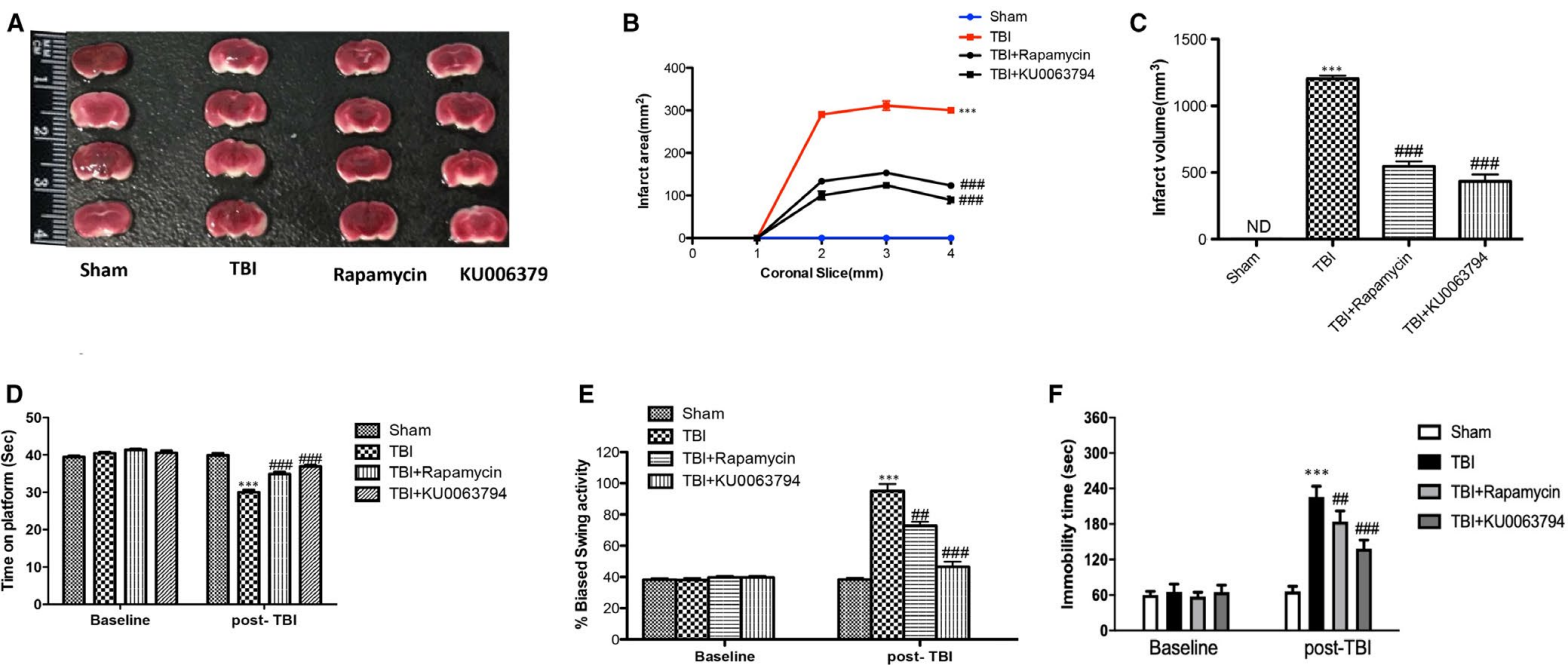
Effect of mTOR inhibitors on brain infarction and on behavioural and neurological function following TBI. TTC staining (brick red) of coronal brain sections (2 mm thick) was performed 24 h after TBI. When compared to intense TTC staining in brains from sham‐operated mice, brains from TBI mice had less TTC indicating greater levels of lesioned area (unstained regions) (A). Lesioned area (B) and volume (C) in rapamycin and KU0063794 treated mice were significantly reduced compared to TBI group. Moreover, significant impairments in motor and neurological deficits were observed in TBI‐injured mice, as revealed by shortened time spent on rotarod (D), augmented biased swing activity (E) and increased immobility time (F). On the contrary, treatment with rapamycin and KU0063794 significantly improved motor function (D and E) and reduced neurological deficits TBI‐associated (F). Data are means ± SEM of 10 mice for each group (three slices per animal). ***P <.001 vs Sham; ^##^ *P* <.01 vs TBI; ^###^ *P* <.001 vs TBI

TTC staining has been performed to evaluate the effects of mTOR inhibitors on brain infarctions in the TBI, revealing an increased necrotic tissue area in TBI mice compared to sham group (Figure 1A); treatments with rapamycin and KU0063794 significantly attenuated the lesion area (Figure 1A) following TBI. Furthermore, the infarction area and infarct volume were significantly reduced after treatment with KU0063794 (Figure 1B,C, respectively). Also, we performed EBST, rotarod test and TST to evaluate motor functions. Mice subjected to CCI showed a variety of impairments in locomotor tasks as shown in Figure 1D,E. KU0063794 treatment more efficacy than rapamycin group improved latency compared with TBI group (Figure 1D,E, respectively). Additionally, as neurological deficit, increased depression and anxiety are often reported by individuals with TBI,^23^ TST was performed to evaluate the neurological function. Treatment with KU0063794, more efficacy than rapamycin, leads to a significant reduction of immobility time in TBI‐injured mice (Figure 1F), compared with TBI‐vehicle group (Figure 1F).

### 3.2 Neuronal injury evaluation after TBI

**FIGURE 2:**
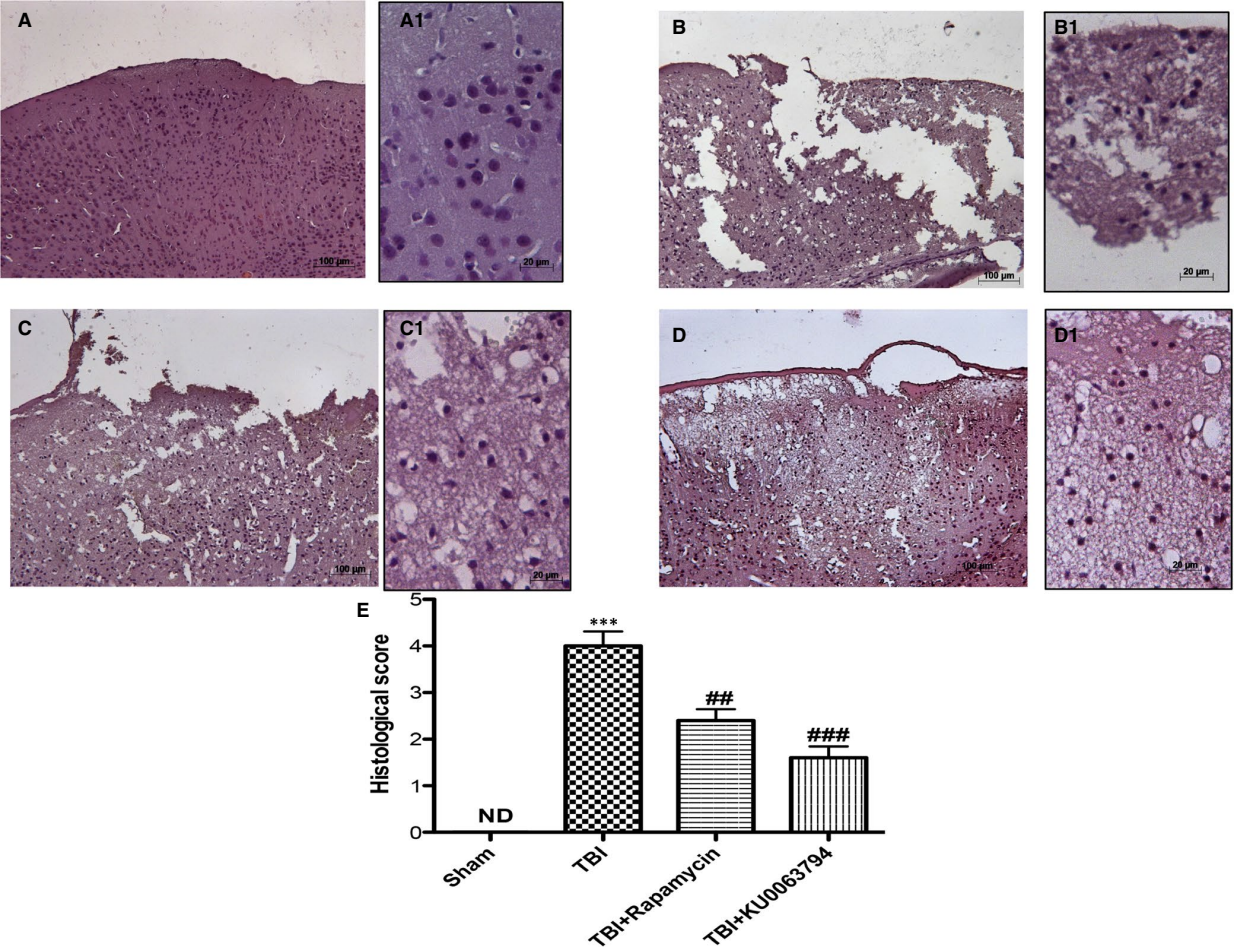
Effects of Rapamycin and KU0063794 treatment on histological alterations after TBI. A histological examination of brain sections showed tissue disorganization and cell infiltration in TBI injured mice (B and B1, see histological score E) respect to intact tissue structure observed in control mice (A and A1, see histological score E). A significant protection from the TBI was apparent in rapamycin and KU0063794‐treated mice (C, C1 and D, D1, see histological score F). The figures are representative of at least three experiments performed on different experimental days. Data are means ± SEM of 10 mice for each group. One‐way ANOVA test (*P* <.05) followed by Bonferroni post hoc test for multiple comparisons. ***P <.001 vs Sham; ^##^ *P* <.001 vs TBI; ^###^ *P* <.001 vs TBI. ND, not detectable

To evaluate the contusion areas after TBI, histological examination by haematoxylin and eosin (H&E) staining was performed, revealing an important tissue disorganization and white matter alteration in the brain parenchyma of TBI mice compared to sham animals (Figure 2A,B, respectively, see histological score 2E). Indeed, KU0063794 treatment significantly decreases the severity of damage more effectively than rapamycin (Figure 2C,D, respectively, see histological score analysis 2E).

### 3.3 KU0063794 modulates NF‐κBp65 pathway after TBI

**FIGURE 3:**
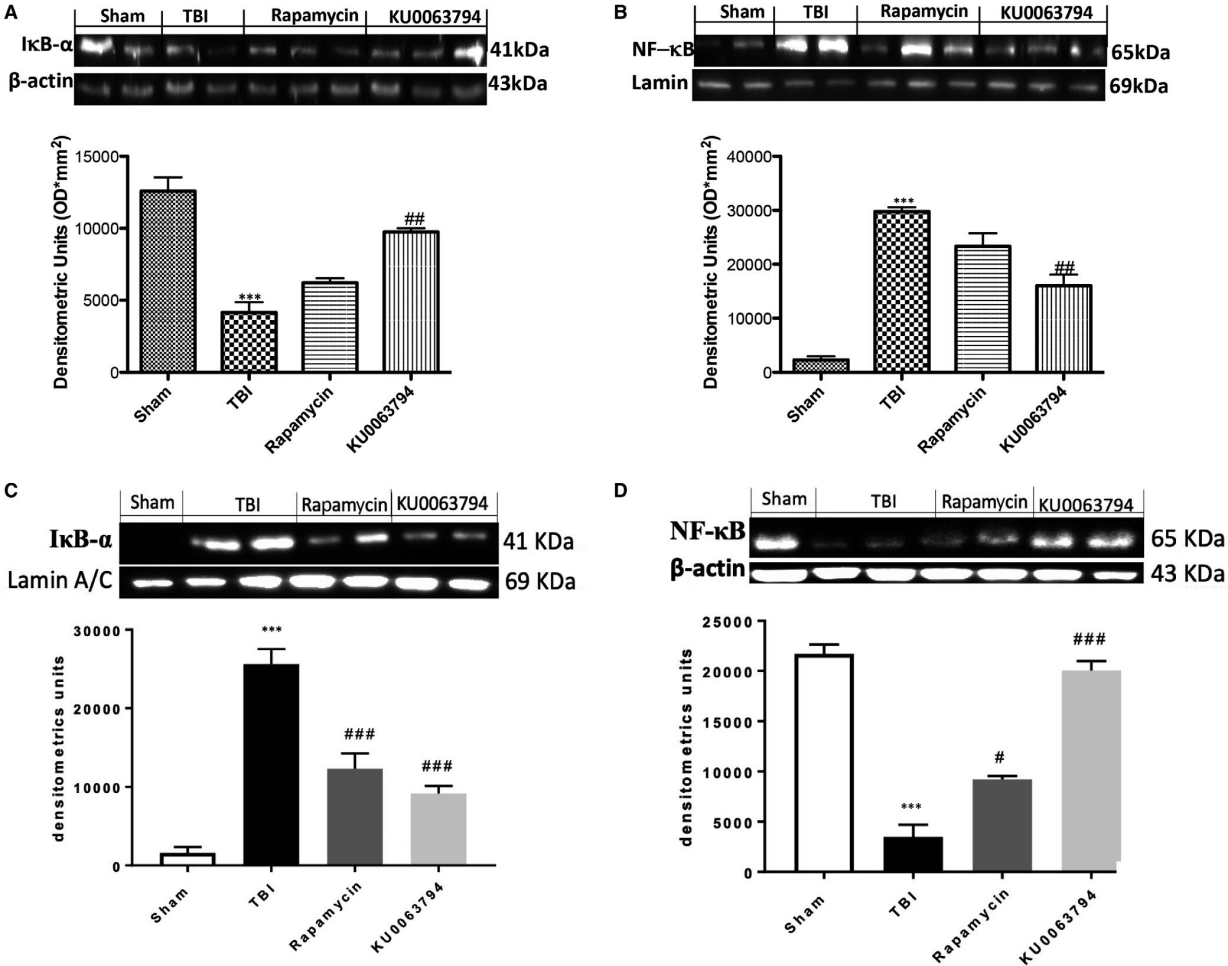
Effects of chronic TBI on Nuclear factor κB (NF‐κB) pathway and pro‐inflammatory enzymes. Degradation of IκBα was significantly increased after TBI (A, A1). Also, TBI resulted in enhanced nuclear translocation of p65 (B, B1), whereas rapamycin and KU0063794 treatment significantly restored IκBα levels (A, A1) and reduced NF‐κBp65 expression (B, B1). IκBα expression was detected in cytosolic fraction, while NF‐κBp65 was detected in nuclear fraction. Data show one representative blot from three independent experiments with similar results. Data are expressed as Mean ± SEM from N = 10 mice/group. ***P <.001 vs sham; ^#^ *P* <.05, ^##^ *P* <.01 and ^###^ *P* <.001 vs TBI

To better understand the role of neuroinflammation in the progression of TBI, we evaluated the NF‐κB pathway by Western blot analysis. Our results showed a significant decrease of IκB‐α expression in TBI‐injured mice (Figure 3A,A1) compared with sham group (Figure 3A,A1), while KU0063794 treatment, more than rapamycin, restored TBI‐induced IκB‐α degradation (Figure 3A,A1). The reduced expression of IκB‐α in nuclear fraction of control group (Figure 3C,C1) and the opposite increase in TBI‐injured group (Figure 3C,C1) confirmed the KU0063794 prevention of IκB‐α cytosolic degradation.

Moreover, KU0063794 treatment considerably decreased the levels of NF‐κBp65 more effectively than rapamycin (Figure 3B,B1), compared to the increase provoked by TBI (Figure 3B,B1); instead to confirm the nuclear translocation of NF‐κBp65, we performed an evaluation of NF‐κBp65 in cytosolic fraction of the samples (Figure 3D,D1).

### 3.4 KU0063794 and rapamycin reduced inflammatory response induced by astrogliosis and microgliosis

To evaluate the anti‐inflammatory and neuroprotective effect of KU0063794 and rapamycin treatments, we performed immunohistochemistry staining for TNF‐α and IL‐1β.

**FIGURE 4:**
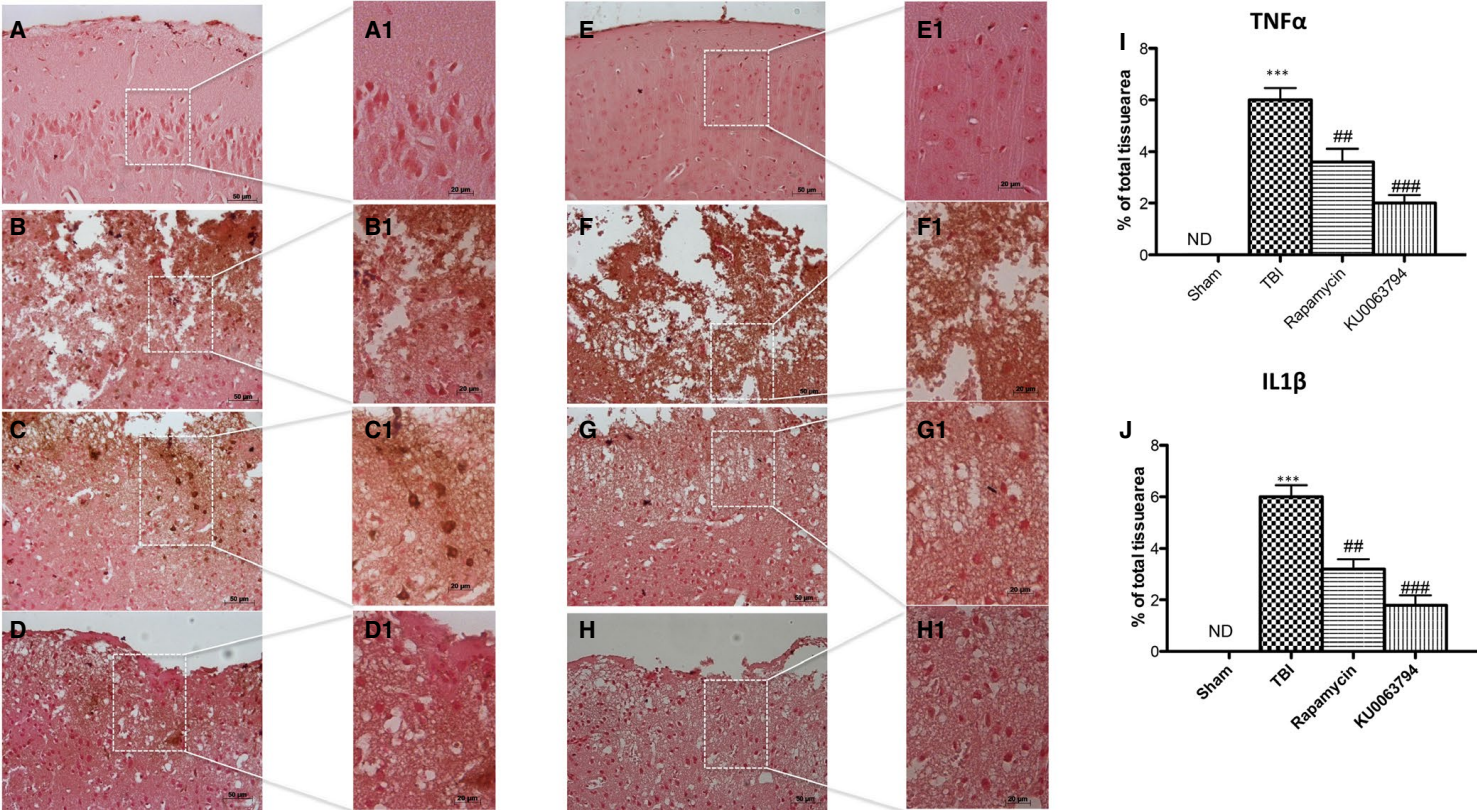
Effect of rapamycin and KU0063794 on proinflammatory cytokines expression following TBI. When compared to sham (A, A1 and E, E1 respectively; see graphs I and J), increased TNFα and IL‐1β (B, D) expression was apparent 24 h following TBI (B, B1 and F, F1, respectively; see graphs I and J). Administration of rapamycin attenuated the expression of both cytokines (C, C1 and G, G1, respectively; see graphs I and J). Moreover, KU0063794 treatment significantly reduced TNFα (D, D1, see graphs I and J) and IL‐1β expression (H, H1, see graphs I and J). The figures are representative of at least three experiments performed on different experimental days. Data are expressed as Mean ± SEM from N = 10 mice/group. One‐way ANOVA test (*P* <.05) followed by Bonferroni post hoc test for multiple comparisons. ***P <.001 vs sham; ^##^ *P* <.01 vs TBI ^###^ *P* <.001 vs TBI. ND, not detectable

We demonstrated that both TNF‐α and IL‐1β levels were significantly increased after TBI (Figure 4B,F, respectively; see graphs I and J, respectively) compared with control group (Figure 4A,E, respectively; see graphs I and J, respectively). KU0063794 and rapamycin treatments significantly reduced TNFα (Figure 4C,D, respectively; see graphs I and J, respectively) and IL‐1β (Figure 4G,H, respectively; see graphs I and J, respectively) production, stimulated by TBI‐induced microgliosis, with a great trend of protection with KU0063794 treatment. These results were confirmed by immunofluorescence analysis, as shown in Figures S1 and S2.

**FIGURE 5:**
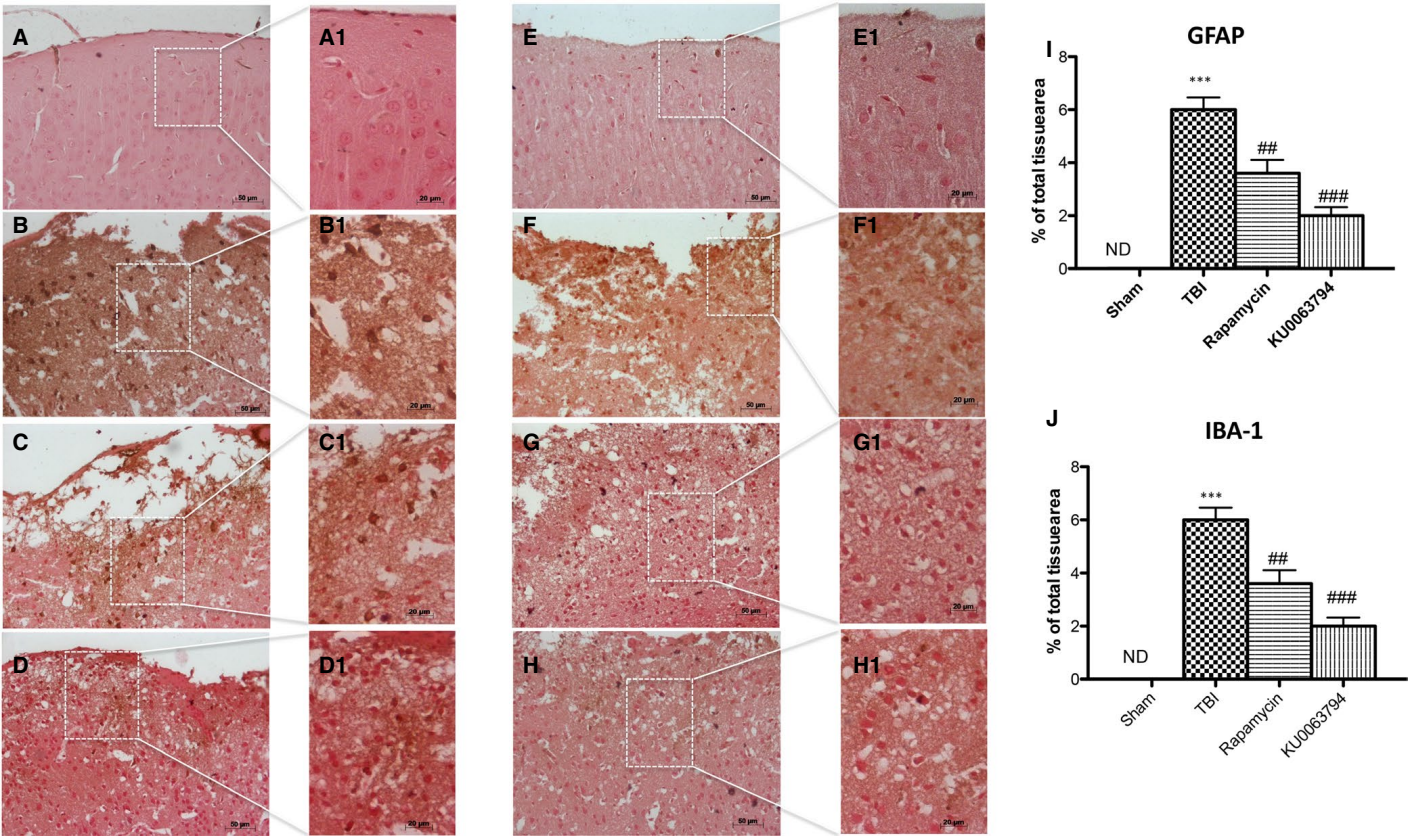
Effect of Rapamycin and KU0063794 on astrocytes and microglia activation following TBI. A remarkable increment in microglia and astrocytes activation was observed by immunostaining for GFAP and Iba1, respectively, markers for astrocytes and microglia activation. In TBI‐injured animals, there was a notably increment in the positive staining for both GFAP and Iba1 (B, B1 and F, F1, respectively; see graphs I and J) compared to sham group (A, A1 and E, E1, see graphs I and J). Treatment with rapamycin and much more with KU0063794 reduced the activation of both astrocytes (C, C1 and G, G1, respectively; see graphs I and J) and microglia (D, D1 and H, H1, respectively; see graphs I and J). The figures are representative of at least three experiments performed on different experimental days. Data are expressed as mean ± SEM from n = 10 male. One‐way ANOVA test (*P* <.05) followed by Bonferroni post hoc test for multiple comparisons. ***P <.001 vs sham; ^##^ *P* <.01 vs TBI ^###^ *P* <.001 vs TBI. ND, not detectable

Microgliosis and astrogliosis processes, meant as astrocytes and microglia activation, are a key component of the pathological onset and progression of TBI. Thus, we evaluated, by immunohistochemistry staining, the expression of Iba1 and GFAP, as a marker of microglial and astrocyte activation, respectively. A substantial increase in GFAP and Iba1 expressions (Figure 5B,F, respectively; see graphs I and J) was found in mice subject to TBI compared with control group (Figure 5A,E, respectively; see graphs I and J, respectively), whereas astrogliosis and microgliosis were significantly decreased by KU0063794 (Figure 5D,H, respectively; see graphs I and J, respectively) and rapamycin treatments (Figure 5C,G, respectively; see graphs I and J, respectively), with a higher trend of protection was observed after KU0063794 treatment. These results were confirmed by immunofluorescence analysis, as shown in Figures S3 and S4.

### 3.5 The ROS‐induced lipid peroxidation modulated by KU0063794 and rapamycin after TBI

**FIGURE 6:**
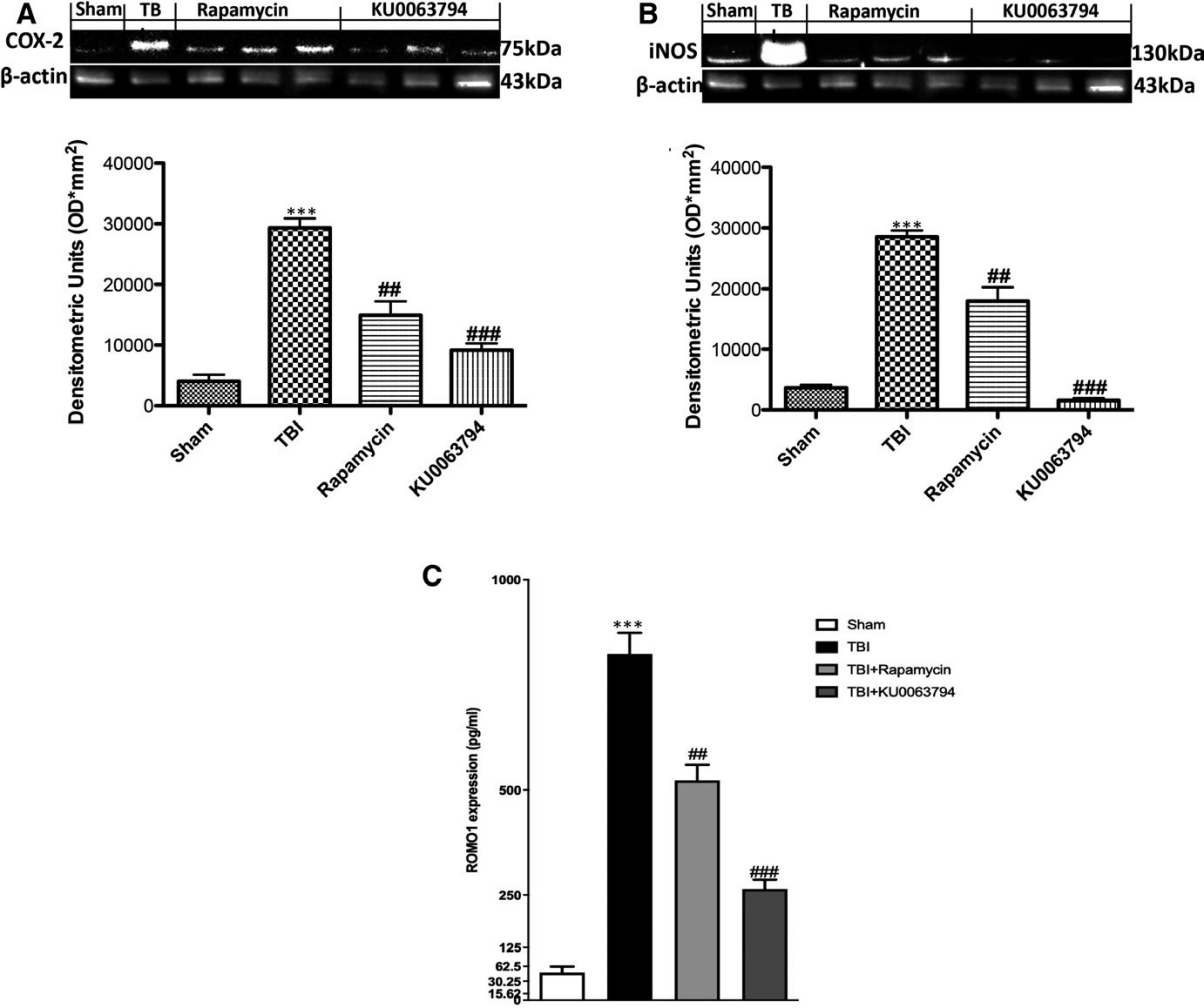
Effects of mTOR inhibition on ROS‐induced peroxidation after TBI. Treatment with KU0063794 significantly reduced ROMO1 expression, compared to TBI‐injured animals (C). A significant increase in COX2 and iNOS was observed in the brain from TBI mice (A, A1 and B, B1, respectively) compared with the Sham mice (A, A1 and B, B1, respectively), whereas rapamycin and KU0063794 treatment significantly reduced COX2 and iNOS expression (A, A1 and B, B1, respectively). The same β‐actin bands were used for COX2 and iNOS. Data show one representative blot from three independent experiments with similar results. Data are expressed as Mean ± SEM from N = 10 mice/group. ^***^ *P* <.001 vs sham; ^##^ *P* <.01 vs TBI; ^###^ *P* <.001 vs TBI

ROS‐induced lipid peroxidation is the most studied mechanism of oxidative damage in TBI.^24^ It is known that ROS modulator 1 (Romo1) is a key mediator for the ROS release,^25^ so to evaluate ROS production, we performed an ELISA kit for ROMO1. A significant increase in ROMO1 expression was observed in TBI‐induced mice compared to control animals (Figure 6C); KU0063794 treatment significantly reduced the ROMO1 expression in samples from TBI‐injured mice, more prominently than treatment with rapamycin (Figure 6C). A key pathway in lipid peroxidation involved activation of inducible nitric oxide (iNOS) and cyclooxygenase‐2 (COX‐2) that we evaluated by Western blot analysis. A considerable increase in COX‐2 and iNOS expression was observed in the brain from mice obtained at 24 hours after TBI (Figure 6A,A1,B,B1), while KU0063794 treatment significantly reduced both expressions more than the treatment with rapamycin (Figure 6A,A1,B,B1).

### 3.6 Effects of KU0063794 and rapamycin treatment on apoptosis pathway after TBI

**FIGURE 7:**
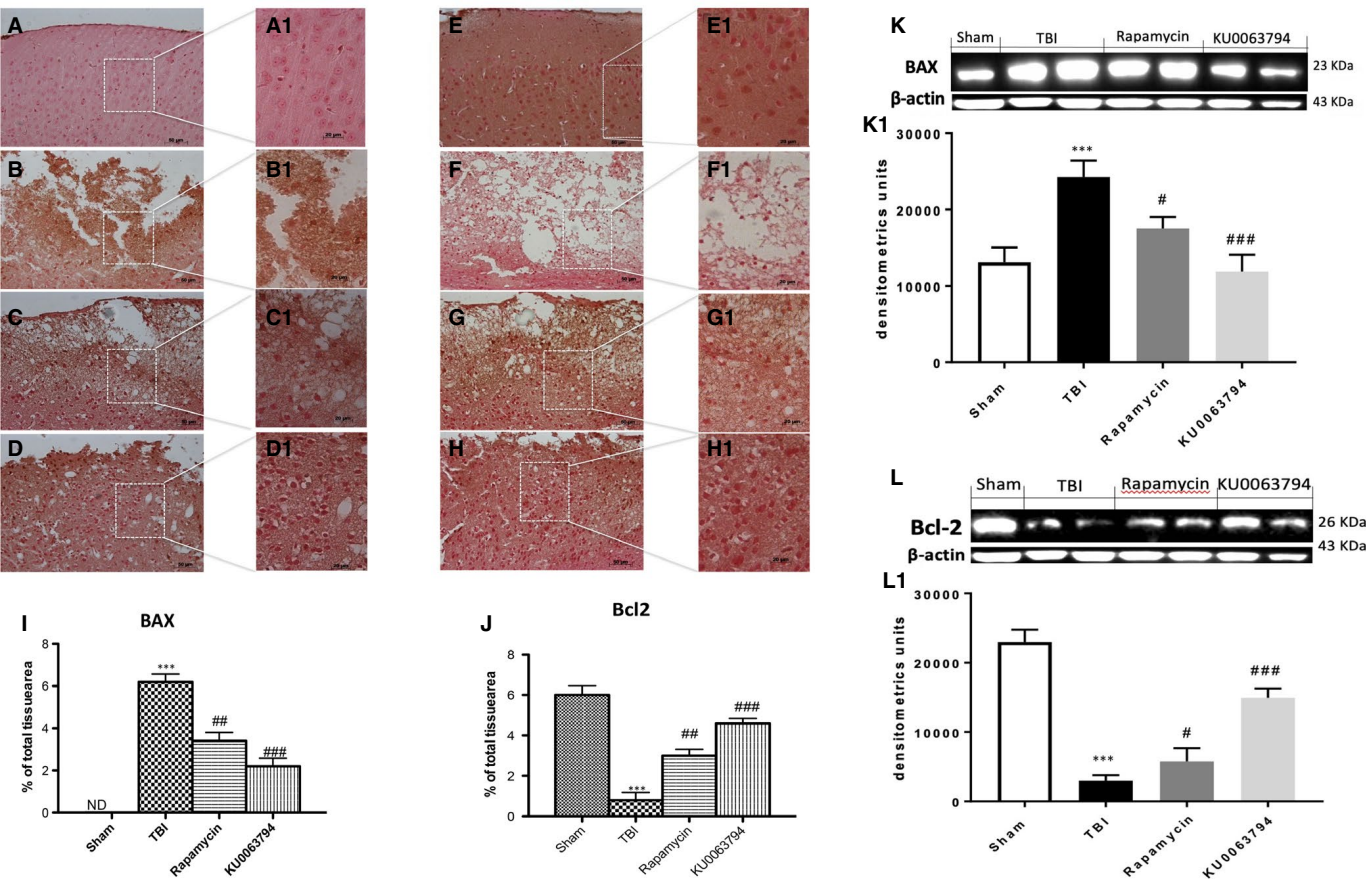
Effects of Rapamycin and KU0063794 on apoptosis after TBI. At 24 h, an increase in Bax expression was observed in TBI‐induced mice (B, B1, see graph I) compared to sham mice (A, A1, see graph I). Treatment with rapamycin reduced the degree of positive staining and expression for Bax (C, C1, see densitometric analysis I), reduction that was more significantly with KU0063794 treatment (D, D1, see graph I). On the contrary, positive staining for Bcl‐2 was significantly reduced in TBI mice compared to control group (F, F1 and E, E1 see graph J), whereas rapamycin and KU0063794 treatment significantly restored Bcl2 staining (G, G1, and H, H1, see graph J). The effects of KU0063794 and rapamycin treatments on Bax and Bcl‐2 expression were also demonstrated by Western blot analysis (Figure K, K1 and L, L1). The same β‐actin bands were used for Bax and Bcl‐2. The figures are representative of at least three experiments performed on different experimental days. Data are expressed as mean ± SEM from N  =  10 mice for each group. One‐way ANOVA test (*P* <.05) followed by Bonferroni post‐hoc test for multiple comparisons. ***P <.001 vs sham; ^##^ *P* <.01 vs TBI; ^###^ *P* <.001 vs TBI

**FIGURE 8:**
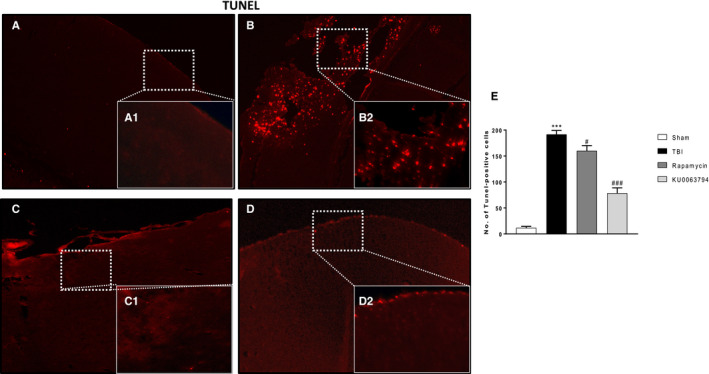
mTORC1/2 inhibition on apoptotic process in TBI by TUNEL assay. The relationship between mTORC1/2 inhibition and apoptosis activation in TBI was demonstrated by TUNEL staining on TBI section after 24 h post‐trauma. Few TUNEL‐positive apoptotic cells were found in sham‐operated mice (A, A1, see graph E), compared with an increase in TUNEL‐positive cells on TBI‐injured mice samples (B, B1, see graph E). KU0063794 and rapamycin treatment significantly reduced the rate of cell apoptosis (D, D1 and C, C, see graph E). The figures are representative of at least three experiments performed on different experimental days. Each data are expressed as Mean ± SEM from N  =  10 mice for each group. One‐way ANOVA test (*P* <.05) followed by Bonferroni post hoc test for multiple comparisons. ***P <.001 vs sham; ^#^ *P* <.05 vs TBI; ^###^ *P* <.001 vs TBI

The role of Bax and Bcl‐2, a pro and anti‐apoptotic factors, respectively, was investigated by immunohistochemical staining. The expression of Bax was substantially increased in the brain subjected to TBI (Figure 7B, see graph I) compared to control mice (Figure 7A, see graph I). On the contrary, KU0063794 and rapamycin treatment prevented TBI‐induced Bax expression (Figure 7C,D; see graph I). The levels of Bcl2 were prompt reduced after trauma (Figure 7B,F; see graph J) and treatments with KU0063794 and rapamycin showed an increase in Bcl‐2‐positive staining (Figure 7G,H, respectively; see graph J) with a better protection by KU0063794 treatment more than Rapamycin. The effects of KU0063794 and rapamycin treatments on Bax and Bcl‐2 expression were also confirmed by Western blot analysis (Figure 7K,K1,L,L1). Furthermore, to fortify the role of KU0063794 and rapamycin on the modulation of apoptosis cell death after TBI, we performed a TUNEL staining on brain section after 24 hours post‐damage. Few TUNEL‐positive apoptotic cells were found in sham‐operated mice (Figure 8A,A1 see graph 8E), compared to a major increase in TUNEL‐positive cells (red dots) on TBI‐injured samples (Figure 8B,B1 see graph 8E). Treatment with KU0063794, better than rapamycin, significantly reduced the number of TUNEL‐positive cells (Figure 8C,C1,D,D1, see graph 8E, respectively).

### 3.7 Effects of KU0064793 and rapamycin on mTOR pathway inhibition

**FIGURE 9:**
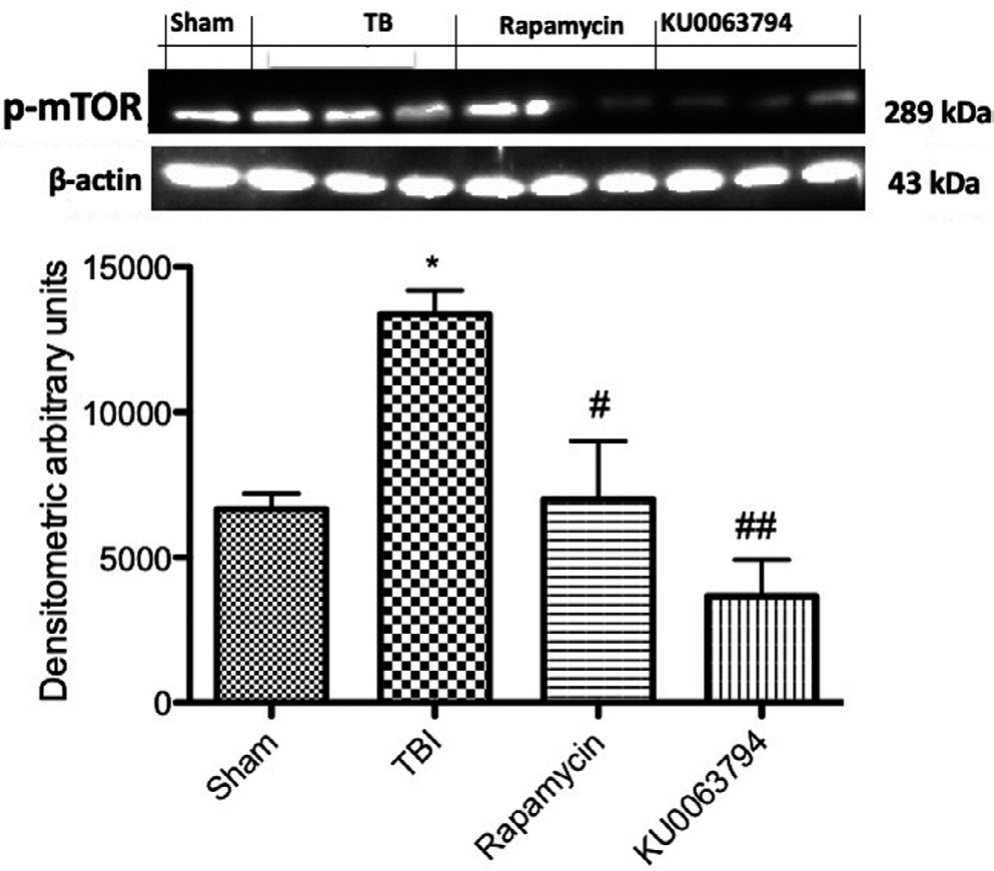
Effects of rapamycin and KU0063794 on p‐mTOR modulation in TBI Western blot analysis on p‐mTOR expression highlighted an increase in samples from TBI‐injured mice (A, A1), compared to control samples (A, A1). KU0063794 and rapamycin treatment significantly reduced p‐mTOR expression (A, A1). The figures are representative of at least three experiments performed on different experimental days. Data are expressed as Mean ± SEM from N  =  10 mice for each group. One‐way ANOVA test (*P* <.05) followed by Bonferroni post hoc test for multiple comparisons. *P <.05 vs sham; ^#^ *P* <.05 vs TBI; ^###^ *P* <.001 vs TBI

To confirm the role of rapamycin to inhibit mTORC1 and evaluate the capacity of KU0064793 to modulate mTORC1/2 activity in TBI model, we performed a Western blot analysis on p‐mTOR expression. A notable increase in p‐mTOR expression was observed in TBI samples, 24 hours after trauma induction (Figure 9A,A1), compared to control samples (Figure 9A,A1). Treatment with KU0064793 significantly reduced p‐mTOR expression (Figure 9A,A1) to a greater extent than Rapamycin treatment (Figure 9A,A1).

## 4 DISCUSSION

Neuroinflammation and microglial activation symbolize the beginning of chronic neurodegeneration and loss of neurological function after trauma.^26^ The secondary injury progresses over the post‐traumatic period as the result of vasogenic and cytotoxic oedema, lipid peroxidation and release of inflammatory regulators.^27^, ^28^, ^29^ The key to developed future neuroprotective approaches, aimed to target neuroinflammation and microglial activation, is to decrease the disadvantageous effects of neuroinflammation promoting beneficial and neurotrophic outcome.^30^ Several recent studies have revealed that phosphorylation of phosphatidylinositol 3‐kinase (PI3K)–Akt–mTOR and its downstream targets (p70S6K, S6 and 4E‐BP1) increased within 30 minutes of a mild injury to the parietal cortex, lasting up to 24 hours; this activation occurs in glial cells as well as in neurons at later time‐point. Previous studies on TBI verified the role of the PI3K/Akt/mTOR pathway in recovery from TBI; in particular, the inhibition of mTOR activation mediated by rapamycin, a strong immunosuppressant, is helpful for ameliorating TBI‐associated symptoms. Autophagy may impair the pathological manifestations of TBI.^31^, ^32^ A mechanism should be developed to increase the therapeutic autophagy, while blocking its harmful side effects.

However, the use of rapamycin has limitations and needs to keep attention in the use; thus, considered that an early intervention post‐TBI could suppress neuronal mTORC activation reducing neuronal damage and prevent glial dysfunction at later stages, we performed a TBI model that reproduces motor deficits and neuron loss that are evinced after trauma, evaluating neuroprotective effects of mTOR kinase KU0063794, that inhibit both mTORC1 and mTORC2 thought phosphorylation of S6K1 and 4E‐BP1.^33^ We supposed that the strategies to target both mTORC1 and mTORC2 may produce better responses after TBI as well as we wonder that KU0063794 has less toxicity of rapamycin and permits a clear interpretation of data. Thus, in our work we assessed the effect of KU0063794 in the control of the inflammatory process associated with TBI, as in the activation of NF‐κB pathway, in the modulation of astrogliosis and microgliosis as well as in the control of pro‐inflammatory mediators’ production. Histological evaluation demonstrated that treatment with KU0063794 determined a reduction of the lesion area and showed a minor morphological modification that are visible following TBI. Further, the treatment of KU0063794 improved motor and neurological behavioural, following the TBI‐associated deficits. Following TBI, the inflammatory condition is a typical response that occurs to the adult mammalian CNS. It is known that reactive gliosis is originated in the surrounding neural tissue and spreads along the edges of the wound by the proliferation and migration of glial cells, and this extended microglial activation at the focal site of injury becomes detrimental over time.^34^ Microglia are rapidly activated at the site of the insult and produce inflammatory mediators such as pro‐inflammatory cytokines that lead to the activation of astroglial cells and neovascularization at trauma sites. Thus, to better recognize whether the mTOR inhibition could modulate the inflammatory process involved in TBI, we evaluated the role of mTOR inhibitors in the control of the inflammatory pathway NF‐κB, clearly demonstrating that that rapamycin and significantly better KU0063794 reduced the translocation of NF‐κB in to the nucleus. NF‐κB consequent translocation in the nucleus determinates the activation and the production of inflammatory factors such as pro‐inflammatory cytokines. Thus, treatment with mTOR inhibitors decreased the inflammatory cytokines, such as TNF‐α and IL‐1β. Therefore, pro‐inflammatory cytokines are synthesized and secreted by astrocytes and microglia; consequently, we investigate the role of mTOR inhibition in modulating astrogliosis and microgliosis by immunostaining for GFAP and IBA1, respectively, markers for astrocytes and microglia activation. Accordingly, we observed that rapamycin and much more KU0063794 significantly reduced the activation of both astrocytes and microglia. Once secreted, these pro‐inflammatory cytokines can bind specific receptors to increase the amount of iNOS and COX2, as well as they can act as molecular inducers of programmed cell death or apoptosis.^35^ We evaluated that mTOR inhibition regulates the expression of iNOS and COX2 that are significantly increased after TBI. COX‐2 and iNOS promote the pro‐inflammatory prostanoids and reactive oxygen species release, contributing to brain injury.^36^ It is generally accepted that one mechanism underlying apoptotic cell death in TBI is a change in the balance between pro‐ and anti‐apoptotic factors towards the expression of proteins that promote cell death.^37^ Therefore, in this study we also observed the role of mTOR signalling on cell death through the modulation of pro‐ and anti‐apoptotic factors; in particular, we showed that treatment with mTOR inhibitors notably reduced apoptosis process, restoring Bcl2 levels. The confirmation of the antiapoptotic role of mTORC1/2 inhibitors is evident in the results obtained and showed for TUNEL staining.

The mTOR signalling pathway is a major player in the control of cell size, dendrite and axon outgrowth during brain insult and repair. Scientific studies have shown that the suppression of mTORC1 by the mTORC1‐specific inhibitor rapamycin after TBI in vivo can have beneficial effects on recovery as measured by extent of tissue damage, motor function, neurological score, and learning and memory tasks in rodent models.^13^, ^38^ In this study, we confirmed the role of rapamycin to inhibit mTOR pathway, highlighting as novelty that treatment with KU0063794 has a greater inhibitory effect on mTOR expression, without the toxicity effects related to rapamycin; therefore, KU0063794 could represent an innovative tool to counteract the mTOR inflammatory alterations associated with TBI. Several inflammatory mediators are involving in systemic tissue damage following acute TBI and recognizing the signalling pathway that could support microglia conserving their regenerative function after injury versus the predominating inflammatory activity, will provide mechanisms in maintaining a healthy brain.

## 5 CONCLUSIONS

Thereby, early intervention with mTOR inhibitors is considerably beneficial to limit tissue damage and improve functional recovery; especially, we described that inhibition of both mTORC1 and mTORC2 resulted more efficacy in reducing neuroinflammation and significantly improving brain function. In conclusion, the understanding of the above pathophysiological mechanisms will undoubtedly help to develop early diagnosis and potential therapeutic strategies and decrease the mortality rate for the TBI patients.

## CONFLICT OF INTEREST

The authors declare that they have no competing interests.

## AUTHORS' CONTRIBUTIONS

SC and EE planned the experiments; IP and MC prepared the manuscript and analysed the results; GC and ML performed experiments; MC and AF performed the histochemical; and SC and AA performed biochemical analysis.

## ETHICS APPROVAL AND CONSENT TO PARTICIPATE

Not applicable.

## Supporting information

Fig S1

Fig S2

Fig S3

Fig S4

## Data Availability

The data sets used and/or analysed during the current study are available from the corresponding author on reasonable request.
