# Supplementary figures and images for: The inhibition of mammalian target of rapamycin (mTOR) in improving inflammatory response after traumatic brain injury
Source: J Cell Mol Med. 2021 Jul 10;25(16):7855–66. doi: 10.1111/jcmm.16702 (PMC8358860; doi:10.1111/jcmm.16702)

IL-1 $\beta$

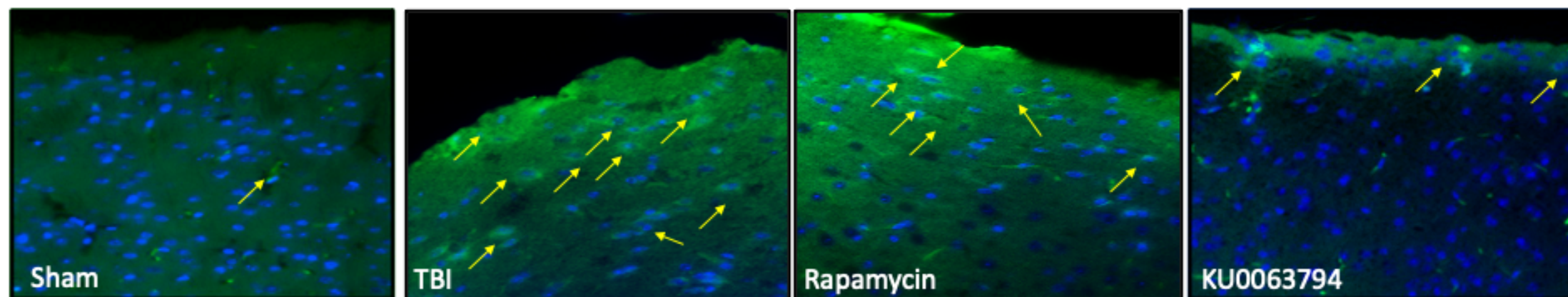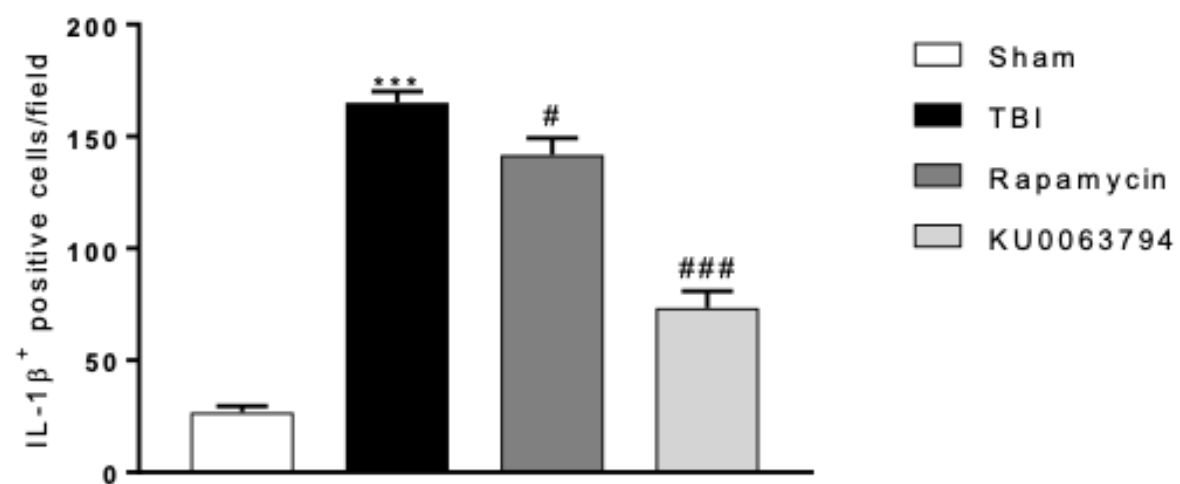

Supplementary Figure 1

Supplement: Supplementary file 1 — Fig S1 [file JCMM-25-7855-s004.pdf]

TNF- $\alpha$

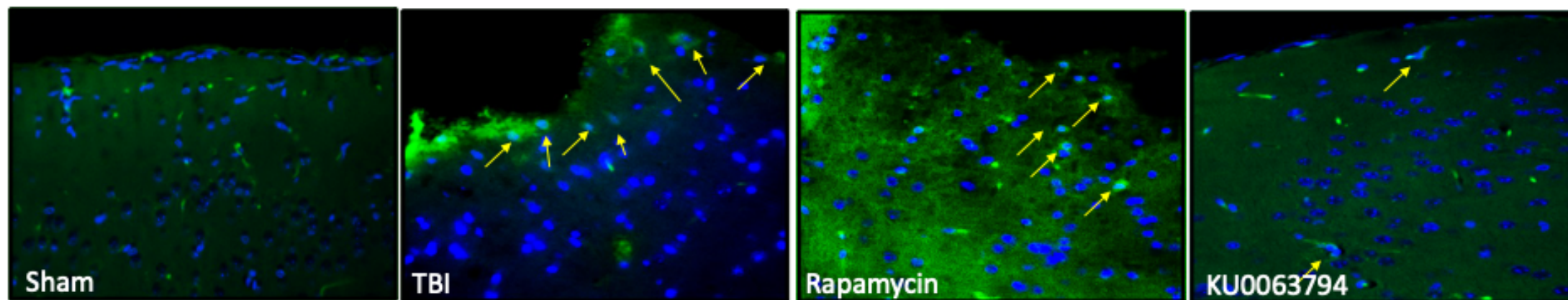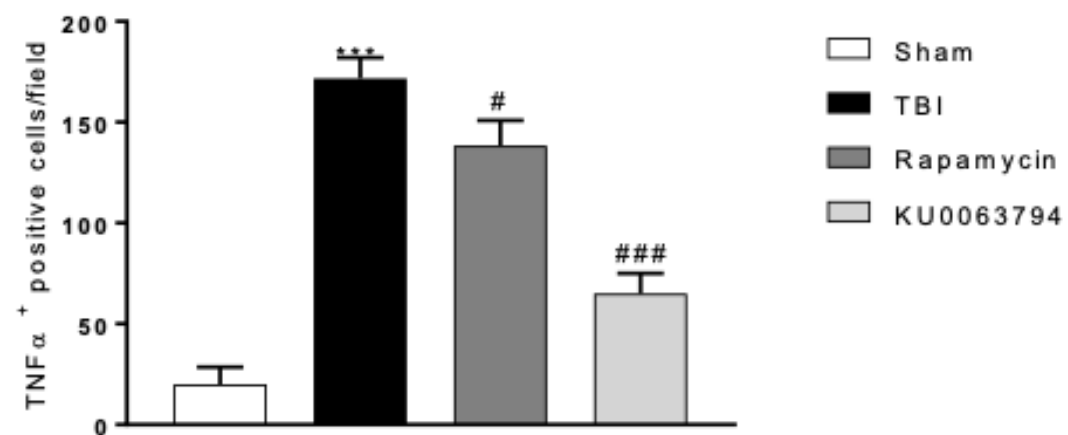

Supplementary Figure 2

Supplement: Supplementary file 2 — Fig S2 [file JCMM-25-7855-s003.pdf]

GFAP

Sham

TBI

Rapamycin

KU0063794

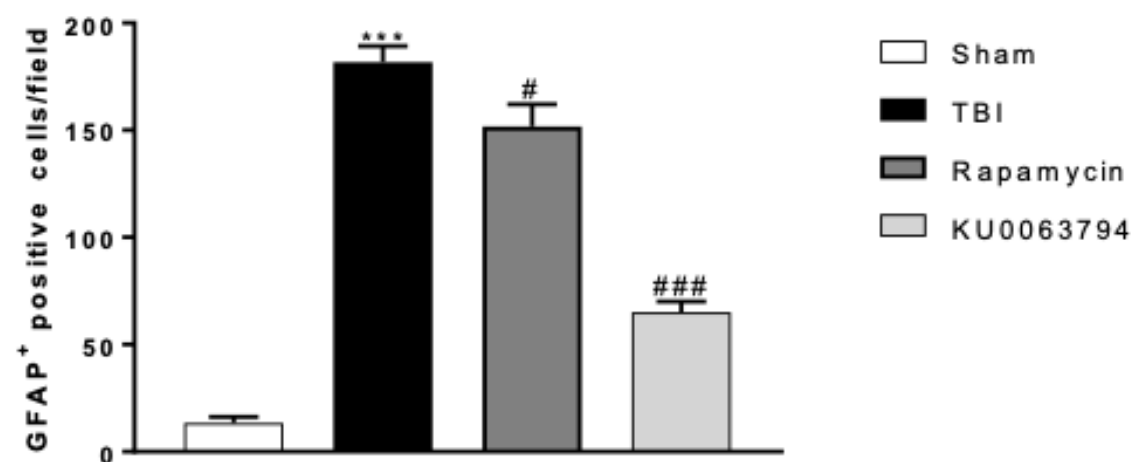

Supplementary Figure 3

Supplement: Supplementary file 3 — Fig S3 [file JCMM-25-7855-s001.pdf]

IBA-1

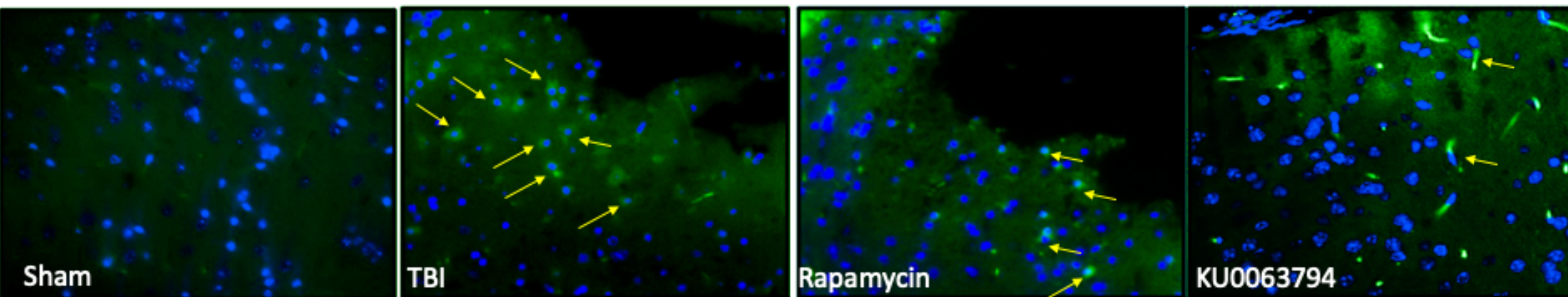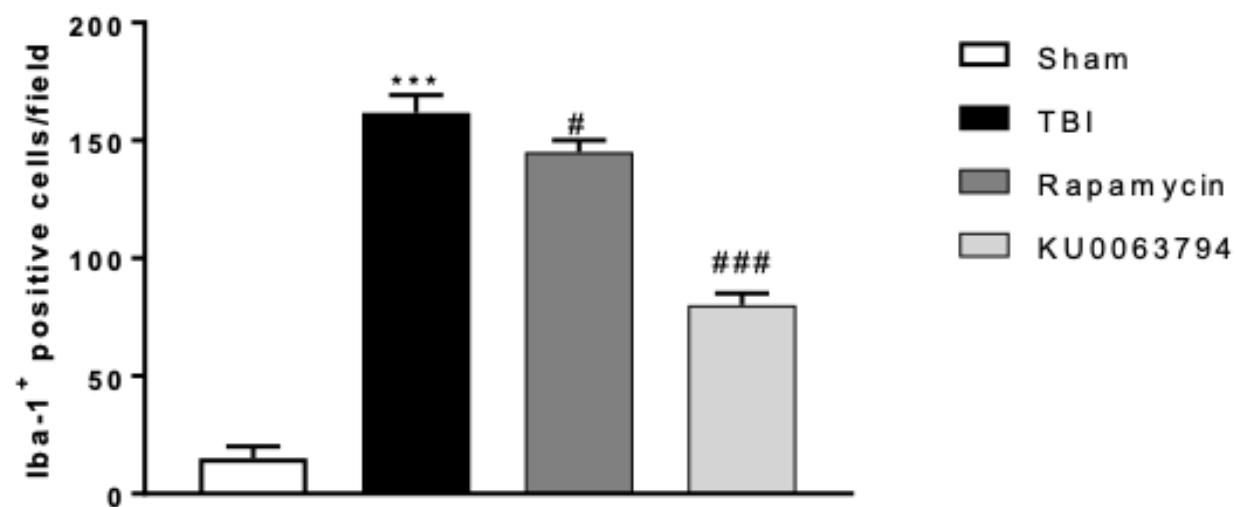

Supplementary Figure 4

Supplement: Supplementary file 4 — Fig S4 [file JCMM-25-7855-s002.pdf]
